# Estimating the effects of vegetation and increased albedo on the urban heat island effect with spatial causal inference

**DOI:** 10.1038/s41598-023-50981-w

**Published:** 2024-01-04

**Authors:** Zachary D. Calhoun, Frank Willard, Chenhao Ge, Claudia Rodriguez, Mike Bergin, David Carlson

**Affiliations:** 1 Department of Civil and Environmental Engineering, Duke University, Durham, NC 27708 USA; 2 Department of Computer Science, Duke University, Durham, NC 27708 USA; 3 Department of Statistics, Duke University, Durham, NC 27708 USA; 4 Rhodes Information Initiative, Duke University, Durham, NC 27708 USA; 5 Division of Natural and Applied Sciences, Duke Kunshan University, Kunshan, 215316 Jiangsu China; 6 Department of Biostatistics and Bioinformatics, Duke University, Durham, NC 27708 USA

**Keywords:** Civil engineering, Climate-change mitigation, Statistics, Projection and prediction

## Abstract

The urban heat island effect causes increased heat stress in urban areas. Cool roofs and urban greening have been promoted as mitigation strategies to reduce this effect. However, evaluating their efficacy remains a challenge, as potential temperature reductions depend on local characteristics. Existing methods to characterize their efficacy, such as computational fluid dynamics and urban canopy models, are computationally burdensome and require a high degree of expertise to employ. We propose a data-driven approach to overcome these hurdles, inspired by recent innovations in spatial causal inference. This approach allows for estimates of hypothetical interventions to reduce the urban heat island effect. We demonstrate this approach by modeling evening temperature in Durham, North Carolina, using readily retrieved air temperature, land cover, and satellite data. Hypothetical interventions such as lining streets with trees, cool roofs, and changing parking lots to green space are estimated to decrease evening temperatures by a maximum of 0.7–0.9   $^{\circ } \hbox {C}$, with reduced effects on temperature as a function of distance from the intervention. Because of the ease of data access, this approach may be applied to other cities in the U.S. to help them come up with city-specific solutions for reducing urban heat stress.

## Introduction

The urban heat island effect refers to the increase in temperature caused by the built environment and human activity^1^. For example, building materials like concrete and asphalt absorb more solar radiation than vegetation, resulting in more heat being released by these materials into the air, and thus, greater ambient temperatures. In some cases, this effect can cause temperatures to be as much as 10   °C warmer than the surrounding countryside^2^. As a consequence of this increased heat, humans are at a greater risk of heat-related illness^3–5^. Moreover, elevated temperatures raise cooling demand to keep indoor environments comfortable. This often means more expensive and greater greenhouse gas emitting energy production to satisfy demand^6^. Thus, to decrease energy consumption and prevent heat-related illness, the urban heat island effect should be mitigated^7^.

Several mitigation strategies have been explored to reduce the urban heat island effect. Of these strategies, cool roofs and urban greening are the most well-studied^8^. The cool roof strategy refers to the use of highly reflective coatings on roof surfaces to increase albedo, or the percentage of incoming solar radiation that is reflected^9^. Cool roofs decrease both the heat released by the building into the surrounding environment, as well as the energy required by the building to maintain comfortable thermal conditions^10^. Urban greening refers to the strategy of increasing the amount of vegetation in cities. Like cool roofs, vegetation increases albedo. Moreover, increased vegetation leads to greater evapotranspiration, which redirects energy from solar radiation to be used for evaporating water instead of heating the air^11^.

Despite this research, predicting the efficacy of any single intervention to reduce the urban heat island effect remains a challenge. This is because the effects of interventions like cool roofs or urban greening depend on where and to what extent they are applied. As a result of this dependence, research into the efficacy of interventions is often restricted to case studies that look at a single city, at a single point in time. Furthermore, because these case studies are often performed at the city scale, they fail to provide insight into the neighborhood-level benefit that interventions could have on lowering the urban heat island effect. For example, one study found that greater than 95% of roofs must be converted to cool roofs to realize urban heat island reductions of 0.5  $^\circ$C across their city^9^. However, the urban heat island effect is not homogeneous at the city scale; temperatures can vary significantly even at the neighborhood-to-neighborhood or city-block scale. As such, we can expect that some neighborhoods will disproportionately benefit from urban heat island mitigation.

To model urban heat islands at higher resolution, two approaches are often taken: a computational fluid dynamics (CFD) approach, and an urban canopy model (UCM) approach. In the CFD model, advection (i.e., heat transfer via wind) is modeled so as to better understand the impact of urban form on the horizontal movement of air. In the UCM approach, urban features such as buildings, concrete, and asphalt can be represented by their thermal properties to produce a model that focuses on how these features impact energy exchange within the urban canopy. However, applying these physics-based approaches may not always be feasible , as CFD models can be too computationally intensive to model micro-scale temperature variability, and the UCM approach is dependent on a large number of modeling assumptions, requiring a high degree of expertise to implement^12^. Due to these barriers, urban heat island researchers often resort to black box models to predict urban heat island intensity. These so-called black box models apply machine learning techniques to vast amounts of data with the objective of maximizing predictive power, while often sacrificing the ability to understand methods for intervening^13^.

A model that is both interpretable and that accurately predicts urban heat island intensity is needed to allow stakeholders to plan interventions to reduce the urban heat island effect. An ideal approach will use readily retrieved data sources, so it may be applied to any city to understand its unique urban heating characteristics. It will also use modeling techniques that require little technical expertise in urban heat modeling. That is, the model will not need technical expertise to be adapted to new locations. Lastly, we believe an optimal methodology will focus on measuring the impact of interventions on air temperature, rather than on land surface temperature. A large portion of urban heat island studies focus on modeling land surface temperature as this data is readily retrieved using satellite imagery. However, land surface temperature is not always the most reliable indicator of the impact of urban heat on human comfort^14,15^. Thus, an ideal model will estimate the effects of interventions on air temperature, which is directly related to human comfort.

To understand how we might achieve such a model, we look to recent innovations in the field of spatial causal inference. Specifically, we explore how the problems of interference and unobserved confounding may be overcome to obtain useful treatment effects for interventions of interest. Such problems are often assumed non-existent in traditional causal inference. We explain why they inherently exist in the context of urban heat island modeling, and propose solutions for overcoming them. We then construct a model around the goal of modeling treatment effects for cool roofs and vegetation. We demonstrate that this model can be developed using already existing surface air temperature data, land cover data, and satellite data, which means that it may be readily applied to a large number of cities in the United States.

In sum, the contributions of this paper are three-fold. First, we demonstrate how spatial causal inference techniques can be applied in the context of urban heat islands. Second, we develop a method to deal with the problems of interference and unobserved confounding, both of which make spatial causal inference challenging. Third, we show how our approach can provide estimates of the effects of local interventions on temperature. To do this, we consider three hypothetical interventions: lining streets with trees, replacing a blacktop with a park, and converting a roof to a cool roof.

## Developing the spatial causal inference model

In this section, we briefly introduce fundamental concepts of causal inference. We explain the issues encountered when applying these concepts to our data, then explain our approach to remedying these issues.

### Preliminaries

We provide a brief overview of the fundamental causal inference concepts required to understand our work, following the main points highlighted in^16^. We refer the reader to this review for a more complete introduction to causal inference in the context of spatial statistics.

The goal in traditional causal inference is to estimate the true causal effect that some treatment $A_i$ has on an outcome $Y_i$ for an individual (or unit) *i*. However, we cannot observe the effect of two different treatments (e.g., a treatment and a control) on a unit at once. This presents the fundamental problem of causal inference^17^. To overcome this problem, we create models that, satisfying certain assumptions, allow us to estimate this causal effect. A standard estimand for causal effects is the average treatment effect (ATE), given in Eq. (1) for the binary case of a treatment ($A_i=1$) and a control ($A_i=0$).

1 $$\begin{aligned} ATE = \mathbb {E}[Y_{i}(A_i=1) - Y_i(A_i=0)] \end{aligned}$$

This estimand corresponds to the view of the potential outcomes framework, in which the average treatment effect is the expected difference between outcomes in a treated and an untreated group. Several assumptions are required for this estimand to be valid:

1. *No interference.* It is assumed that treatments are independent of one another and that giving the treatment to one unit *i* has no impact on another unit $i'$.
2. *Consistency.* There is no hidden variability in the treatment. That is, if a treatment *A* is allocated to a group of units, then the treatments given to each unit are the same.
3. *Latent ignorability.* The outcomes *Y* and treatments *A* are conditionally independent given observed confounding variables $\textbf{X}$. In other words, there is no unobserved confounding^18^.

Assumptions 1 and 2 are commonly referred to as the Stable Unit Treatment Value Assumption (SUTVA)^19^. In spatial causal inference, assumptions 1 and 3 are often (and sometimes inherently) broken. In the context of urban heat islands, we may explain the presence of interference by the impact that trees have on temperature. Locally, a tree provides shade and increases the amount of energy used for evaporation rather than heating. Both of these effects will most significantly decrease temperature closer to the tree, with a decayed effect as distance from the tree increases. Thus, to understand the causal effect of planting a tree on temperature, we must estimate the effect as a function of distance, and we must control for interfering effects of neighboring vegetation. Latent ignorability is also readily understood in this context. The effect of a tree on temperature depends on wind. No existing wind data exists at the resolution of data with which we are working. Thus, we have at least one known unobserved confounding variable, and perhaps many more unknowns. We develop solutions for dealing with these broken assumptions in the following sections.

### Dealing with interference

In the absence of interference, and under an assumption of no unobserved confounding variables, we can readily estimate the linear causal effect of a treatment $A_{ij}$ on an outcome $Y_{ij}$ at any point *i*, *j* in a latticed spatial dataset using the model provided in Equation 2:

2 $$\begin{aligned} Y_{ij} = \beta A_{ij} + \mathbf {\gamma } \textbf{X}_{ij} + \varepsilon _{ij} \end{aligned}$$

In which $\textbf{X}_{ij}$ represents observed confounders for which we wish to control, and $\beta$ and $\gamma$ refer to the learned parameters of the model, with $\varepsilon _{ij}$ remaining as a random error term. We bold $\textbf{X}_{ij}$ and $\mathbf {\gamma }$ to indicate that these are vectors comprised of all observed confounding variables and their coefficients. Note that this equation implicitly assumes that the relationship between $A_{ij}$, $X_{ij}$, and $Y_{ij}$ is linear. We maintain this assumption for the purposes of this work and leave non-linear considerations as future work.

However, if we wish to control for interference, then we must look at the effect of surrounding points on $Y_{ij}$. One proposed solution for dealing with interference is proposed in^16^, in which they add two terms to this model: $\bar{a}_{ij}$ and $U_{ij}$, which refers to the interference term and the unobserved spatial confounding term, respectively. Formally, this updated equation is given by Eq. (3). This equation is adapted from its original point-indexed notation to deal with lattice-indexed data, as our final objective is to apply this model to satellite (i.e., gridded/pixelated) data.

3a $$\begin{aligned} Y_{ij}= & {} \beta _0 A_{ij} +\beta _1 \bar{A}_{ij}+ \mathbf {\gamma } \textbf{X}_{ij} + U_{ij} + \varepsilon _{ij} \end{aligned}$$

3b $$\begin{aligned} \bar{A}_{ij}= & {} \sum _{k \ne 0}\sum _{l \ne 0} w(\sqrt{k^2 + l^2})A_{i+k,j+l} \end{aligned}$$

**Figure 1:**
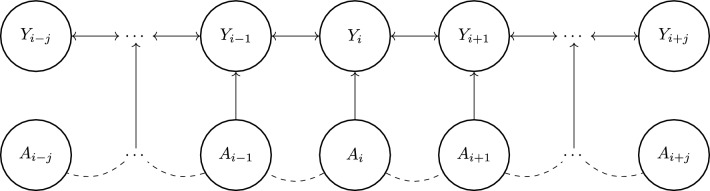
A simple one-dimensional graph for modeling the causal relationship between covariates and temperature. Dashed lines indicate an unobserved confounding relationship and solid lines represent a causal relationship.

In this formulation, a weighting function *w* is introduced that incorporates the effect of treatment levels at surrounding points, and *k*, *l* refer to the pixel distance from the point *i*, *j*, which we convert to the Euclidean distance. This weighting term is defined as a function of the distance from the point of interest, and thus, to adequately control for the effects of neighboring points, this function must be reasonably defined. In theory, the interference level between very distant points could still be considered in $\bar{A}_{ij}$. However, in practice, it suffices to only consider a window size around each point of interest, as we expect the weighting term to become sufficiently small at a far enough distance. For our model, we justify the use of an exponential function through the graphical model in Fig. 1, which represents a simplified one-dimensional case of heat transfer.

### Deriving a weighting function from a simplified graph

Here, we show how the exponential function would arise from a simplified one-dimensional version of the gridded space. A similar argument holds for the full gridded space but is more complex. In a one-dimensional representation of urban heating, we assume that the treatment $A_i$ at any point *i* has a direct effect on the temperature $Y_i$, and that the indirect effect of $A_i$ at neighboring temperature points $Y_{i-1}$ and $Y_{i+1}$ is mediated through the effect on $Y_i$. We reason that this represents the effect of advection on heat transfer.

If we temporarily omit the effect of spatial confounding and random error, then we would represent the graphical relationship in Fig. 1 through the equation:

4 $$\begin{aligned} Y_i = \alpha A_i + \beta (Y_{i-1} + Y_{i+1}) \end{aligned}$$

Where $\alpha$ and $\beta$ are referring to the learned parameters of the model. Under this parameterization, we assume that having a lower or higher temperature at surrounding points is going to have a corresponding effect on the temperature at the point of interest. However, since the goal of our model is to learn the indirect effect of covariates at neighboring points, we would like to obtain a form of the model in which the observed temperatures are removed from the model so that we have an equation for temperature that is completely defined in terms of the covariates. To do this, we can expand Eq. (4) by writing out the equations for $Y_{i+1}$ and $Y_{i-1}$:

5 $$\begin{aligned} Y_{i\pm 1} = \alpha A_{i\pm 1} + \beta (Y_{i} + Y_{i\pm 2}) \end{aligned}$$

When writing out the equations for the temperature at neighboring points, we can start to see the beginning of a recursive relationship. If we substituted this function back into the original model, we would then get a further expanded equation:

6 $$\begin{aligned} Y_{i} = \alpha [A_{i} + \beta (A_{i-1} +A_{i+1})] + \beta ^2 (Y_{i-2} + 2Y_{i} + Y_{i+2}) \end{aligned}$$

Upon continued expansion, a pattern among the coefficients emerges. Interestingly, this pattern is first noticed through the coefficients leading the *Y* terms on the right-hand side of the equation, in which the numbers continue expanding according to the columns of Pascal’s triangle. Thus, the expansion of coefficients that correspond to $A_i$ will take the center column of Pascal’s triangle, so that the coefficient will be of the form $k_0 = 1 + 2\beta ^2 + 6\beta ^4 + 20\beta ^6 +...$. Upon recognizing this pattern, For each term $a_{i\pm j}$, in which *j* refers to the distance from point *i*, the general pattern takes the form of:

7 $$\begin{aligned} k_j = \sum _{n \ge 0} \left( {\begin{array}{c}2n + j\\ n\end{array}}\right) \beta ^{2n+j} \end{aligned}$$

In which $k_j$ refers to that expanded coefficient. Fortunately, this binomial expansion is well known, and it can be shown through a simple application of Wilf’s Snake Oil method^20^ that the closed form is:

8 $$\begin{aligned} k_j = \frac{\beta ^{j}}{\sqrt{1 - 4\beta ^2}}\bigg (\frac{1-\sqrt{1-4\beta ^2}}{2\beta ^2}\bigg )^j \end{aligned}$$

This means that the fully expanded equation for the effect of treatment *A* can be expressed as:

9 $$\begin{aligned} Y_i = \alpha \left[ \frac{A_i}{\sqrt{1-4\beta ^2}} + \sum _{j\ne 0}\frac{\beta ^{|j|}}{\sqrt{1-4\beta ^2}}\bigg (\frac{1-\sqrt{1-4\beta ^2}}{2\beta ^2}\bigg )^{|j|} \bigg (A_{i+j}\bigg )\right] \end{aligned}$$

In which *j* includes all integers. This expansion allows us to notice two important features of this model: the direct effect of a treatment on temperature is dependent on the level of interference (as shown with $\beta$ in the denominator), and the indirect effect of neighboring treatments decreases exponentially as a function of distance. For the purposes of our analysis, this closed-form solution justifies the use of an exponential function for the weighting function originally defined in Eq. (3). Thus, we can define *w* as:

10 $$\begin{aligned} w(j) = \frac{\exp (-j / \ell )}{\sum _{k\ne 0} \exp (-k / \ell )} \end{aligned}$$

In which $w_j$ is normalized by the denominator over the neighboring *k* points so the sum of all weights is equal to 1, and the length scale parameter $\ell$ must be tuned to control for the level of interference. We generalize this finding beyond the one-dimensional case by considering *j* as the Euclidean distance between points. To reduce the computational cost of calculating the weighted interference terms, we constrain the weighting function to only consider a window around each point of interest. Since we are using gridded data, we find that a window size of 51x51 pixels around each point is sufficient before the weights get too small. Since our data is at a 10 m resolution, this corresponds to considering pixels within 250 m of each point.

### Dealing with unobserved confounding

Unobserved confounding shows up in spatial modeling in two scenarios: (1) when there is an unobserved factor that covaries with a treatment, and (2) when there is an autocorrelated spatial error term^21^. In the context of causal inference, unobserved confounding can bias estimated treatment effects. In an attempt to remove this bias, we re-arrange Eq. (3) so that unobserved confounding is a function of the spatial residuals of the treatments and observed confounding variables, given in Eq. (11). We assume that there is still some noise in the unobserved confounding term, so we keep the random error term $\varepsilon _{ij}$.

11 $$\begin{aligned} U_{ij} = Y_{ij} - (\beta _0 A_{ij} +\beta _1 \bar{A}_{ij}+ \mathbf {\gamma } \textbf{X}_{ij}) + \varepsilon _{ij} \end{aligned}$$

We then use this equation to learn an underlying spatial process for the unobserved confounding term as a function of the coordinates, which we parameterize as a Gaussian process. While an in-depth discussion on Gaussian Processes is beyond the scope of this work, we elect to use a Gaussian process because it allows us to explicitly specify a covariance structure for our residuals. For an in-depth overview of Gaussian processes, we refer the reader to^22^. The ability to specify the covariance structure is important so that we can constrain the unobserved confounding term to be smooth enough to only model autocorrelated residuals at a scale larger than that of the covariates. In the context of causal inference, it has been shown that if the unobserved confounding deals with variance at spatial scales greater than those of the covariates and treatments, then we may decrease bias of treatment effect estimates^23^.

This covariance structure is defined through a covariance function, or a kernel, which is just a function that specifies how we expect covariance to behave as a function of distance between points. In the context of our problem, we find that a suitable kernel is an exponential function summed with a dot product function, as this can be parameterized to both prevent overfitting, and to ensure only larger scale variability is modeled by the Gaussian process. We formally define the covariance function in Eq. (12). In this definition, we refer to the two-dimensional coordinates as a vector using $\textbf{s}$, the length scale $\ell$, the inhomogeneity parameter $\sigma _0$, and two constants $\alpha _1$ and $\alpha _2$, which control the strength of each kernel term. Lastly, we indicate the Euclidean distance using $||\cdot ||$.

12 $$\begin{aligned} K(\textbf{s}_i, \textbf{s}_j) = \alpha _1^2 \exp (-||\textbf{s}_i - \textbf{s}_j||/\ell ) + \alpha _2^2 (\sigma _0^2 + \textbf{s}_i \cdot \textbf{s}_j) \end{aligned}$$

We choose an exponential kernel to be consistent with the exponential weighting terms in the regression, which allows us to ensure that the length scales of the kernel are greater than the length scales of the regression terms. We include the dot product kernel to model larger-scale non-stationarity in the data. In other words, we see a gradient in the residuals that appears to be modeled well as a linear function of the coordinates themselves (see Fig. 4b). In the case of modeling urban heat islands, we attribute non-stationarity to weak synoptic scale advection. We treat kernel parameters as hyperparameters, tuned using cross-validation (further details provided in the “Methods” section).

### Modeling temperature with albedo, vegetation, and land cover

Given the theoretical model in Eq. (3) along with the weighting function defined in Eq. (10), we can now formalize the final model used for our analysis. To estimate the causal effects of cool roofs and vegetation as interventions when controlling for the effects of land use, we propose the model in Eq. (13), using the variables *V* for vegetation, *A* for albedo, and $\textbf{X}$ for land cover. We use $\beta$ for coefficients for the causal variables of interest, and $\gamma$ for the coefficients corresponding to the observed confounding variables.

13a $$\begin{aligned} Y_{ij}= & {} \beta _1 V_{ij} + \beta _2 \bar{V}_{ij} + \beta _3 A_{ij} + \beta _4 \bar{A}_{ij} + \mathbf {\gamma }\mathbf {X_{ij}} + U_{ij} + \varepsilon _{ij} \end{aligned}$$

13b $$\begin{aligned} \bar{V}_{ij}= & {} \sum _{k \ne 0}\sum _{l \ne 0} w_{V}(\sqrt{l^2 + k^2})V_{i+k,j+l} \end{aligned}$$

13c $$\begin{aligned} \bar{A}_{ij}= & {} \sum _{k \ne 0}\sum _{l \ne 0} w_{A}(\sqrt{l^2 + k^2})A_{i+k,j+l} \end{aligned}$$

We note that since there are two causal covariates of interest, we require two weighting functions. As such, two separate length scales will need to be fine-tuned during training. By learning two distinct length scales, we can learn at what spatial scales these two covariates affect their surroundings. Additionally, land cover was initially encoded as a one-hot vector corresponding to the land cover classes defined by the National Land Cover Database. We attempted to treat this as a causal variable with an additional spatial term. However, we found that treating this variable as a weighted average of the classes within the window around each data point resulted in approximately equal performance. We theorize that this is because a small subset of the land cover classes account for most of the classes co-located with the temperature data (see Fig. 3), and that the length scales at which interference occurs in the data is large enough such that the exponential weighted average ends up being similar to a simple weighted average of the pixel classes anyways. Furthermore, land cover changes may be considered less realistic interventions than the interventions of increasing vegetation and albedo. This allows us to simplify the causal interpretation of Eq. (13).

The regression terms of Eq. (13) (i.e., all terms besides $U_{ij}$) are fit using an $L_2$ penalty, which is commonly used in ridge regression. Because there is multicollinearity across the covariates, this penalty ensures that the strongest relationships are captured by the model. This provides one source of bias in our approach, as the coefficients will be shrunk by the penalty. Due to this shrinkage, our model may provide conservative estimates of treatment effects. We describe the optimization procedure in the “Methods”.

### Summary of approach

**Figure 2:**
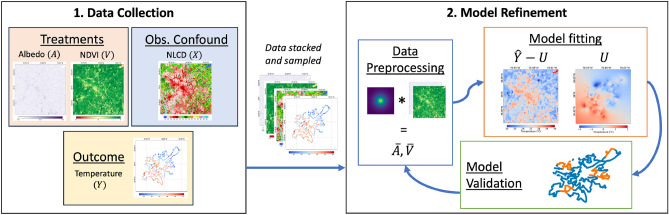
Summary of approach. We first collect data on the treatments of interest (*A* and *V*) and outcome (*Y*), as well as confounding variables that also impact the outcome (*X*). We stack and sample this data to create the dataset used for model fitting. Since the temperature data was collected via vehicular traversal through the study area, this process entails sampling each point along the traversal, then selecting the 51 $\times$ 51 pixel window from the treatments and confound that are centered on this point. We start refining the model by preprocessing the data. The 51 $\times$ 51 pixel windows corresponding to treatments are multiplied by a weight matrix that weights pixels closer to the center higher than points further away, as dictated by a length scale parameter on the weight matrix definition. This product provides a spatially-averaged value that estimates the effect of neighboring pixels on the temperature at the center of the sample, denoted by $\bar{A}$ and $\bar{V}$. We select the center pixels from the treatments to capture the direct effect of treatments on the outcomes. The preprocessed dataset used for model fitting is then a set of tuples comprised of spatially weighted albedo, center pixel albedo, spatially weighted vegetation, center pixel vegetation, average land cover class, and temperature. Our model fits the data with a regression, then adjusts the regression with a Gaussian process that models unobserved confounding (denoted *U*). This provides us with an estimate of temperature ($\hat{Y}$) with and without the effect of unobserved confounding. The model is then validated using a cross-validation strategy that segments out samples from the traversal into blocks, with a subset of blocks held-out from model fitting to evaluate model performance. We update the weight matrix and model parameters to maximize the performance of the model on this held-out set. The best model is used to estimate effects of interventions on temperature.

We summarize the data collection and model fitting procedure in Fig. 2, with further details provided in subsequent sections.

## Data collection

**Figure 3:**
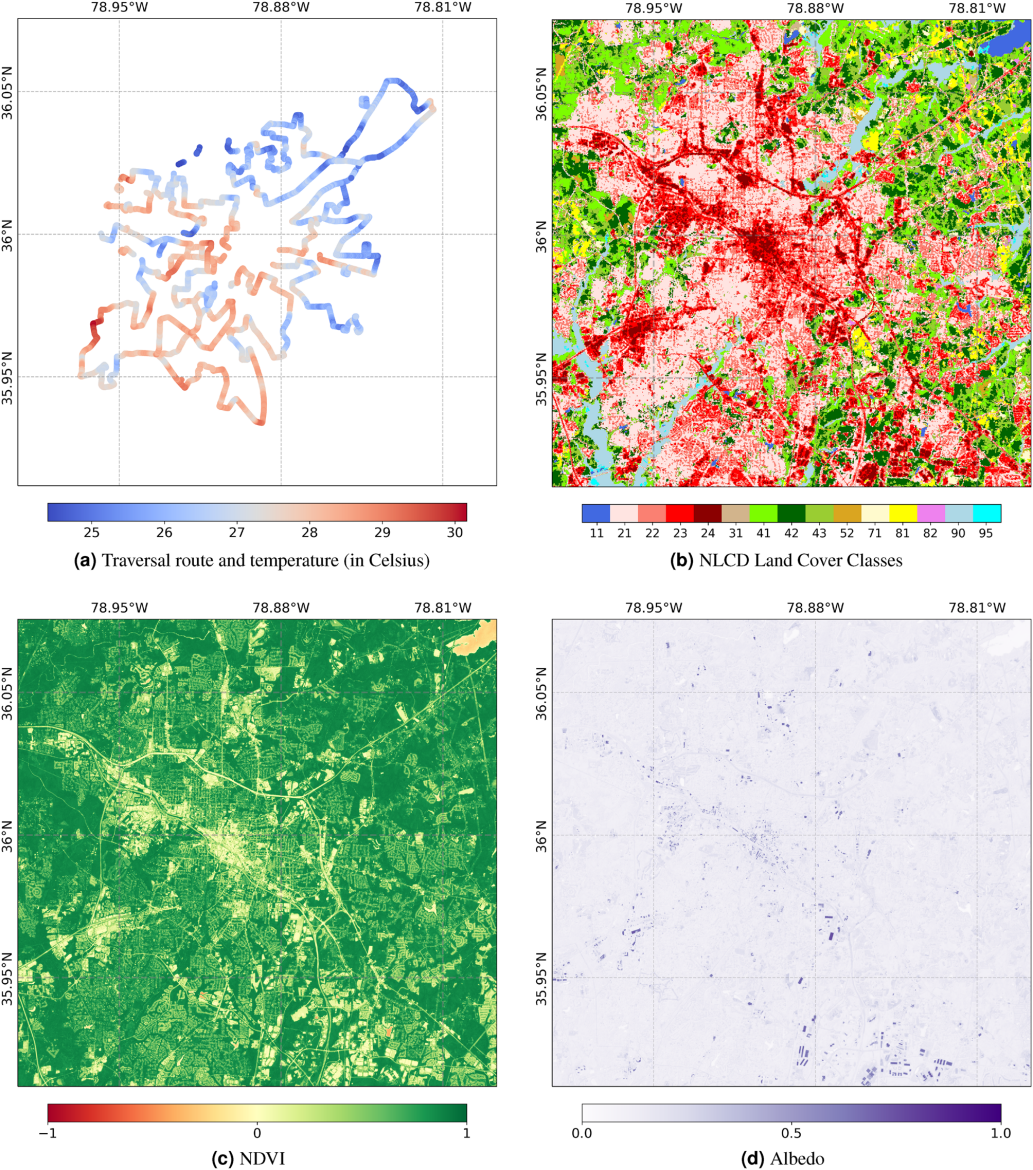
Datasets used for analysis. Land cover classes are provided in Table 1.

To demonstrate our approach, we apply this model to Durham, North Carolina, on a typical summer day (July 23, 2021). Four datasets are used within this model: a surface air temperature dataset, a derived albedo measurement dataset (using Sentinel-2), the normalized difference vegetation index (NDVI, calculated using Sentinel-2), and the National Land Cover Database (NLCD). These datasets may be visualized in Fig. 3, with further details provided below on data collection.

### Temperature dataset

We use temperature data collected through NOAA’s (National Oceanic and Atmospheric Administration) urban heat island mapping campaign for our model, which uses the approach described in^24^, with more specific details for the Durham campaign available at^25^. This data source is chosen because it provides surface air temperature at high spatial resolution, on representative days for the selected urban area. To collect this data, volunteers attached temperature sensors to their vehicles and drove around around a traversal path at three periods of time throughout the day. We used the data collected during the evening time frame, as this is when temperature differences were greatest. This data collection method was first pioneered in^26^, and is commonly used in urban heat island research.

A large body of urban heat island research uses Land Surface Temperature (LST) to characterize urban heat island intensity^27,28^. LST data is often preferred because it is easily collected via satellite products, enabling data collection for large areas over long time periods. In this study, we opt instead for an air temperature dataset, as this is more representative of the urban heat island’s effect on human health and energy consumption^29^. Moreover, because this dataset is collected as point data, we are able to calculate spatial effects at a higher resolution (10m) than the frequently used LandSat LST resolution (30m). The point data is collected at higher than 10 meter resolution, and we used the GDAL utility to convert the point data to a rasterized format^30^. If there were more points in each 10-m by 10-m pixel, we averaged the temperature values. This data conversion resulted in a 2253-by-2307 pixel image of the data, with 12,448 temperature measurements. This image size was selected to have a 1 km (100 pixel) buffer around each measurement. The accompanying land cover, albedo, and vegetation data was subset to match these dimensions.

### Land cover

The National Land Cover Database was selected for use in this study, for its high quality and ease of availability. We selected the data for 2021, the same year the temperature data was collected^31^. We then upsampled the dataset from its original resolution of 30 meters to match the albedo and vegetation resolution at 10 meters, using the GDAL utility, with the nearest neighbor upsampling method.

The Local Climate Zone (LCZ) classification scheme for land cover is often recommended for understanding the urban heat island effect^32^. We choose to use NLCD instead of LCZ because NLCD is readily downloaded for the entire United States, making it an easier to use data source. While LCZ data can be found online, it is either at low resolution (> 100 m) or limited to specific sites. In contrast, NLCD is consistently updated for the entire United States at 30 meter resolution. Empirically, we find that the LCZ classification scheme tends to correlate with the NLCD classification scheme for our areas of interest. Thus, we expect the LCZ system to offer few advantages given its lower availability.

### Albedo

We calculated albedo using the method defined in^33^, with Sentinel-2 surface reflectance data. The Sentinel-2 data was collected using Google Earth Engine, using the least cloudy data within 2 months of the date of the temperature collection^34^.

### Vegetation

The Normalized Difference Vegetation Index (NDVI) is a commonly used satellite-derived metric for quantifying vegetation in an area, calculated using the near-infrared (NIR) and red bands of a satellite image. The calculation is defined as:

14 $$\begin{aligned} \text {NDVI} = \frac{\text {NIR} - \text {Red}}{\text {NIR} + \text {Red}} \end{aligned}$$

The same Sentinel-2 image collected for the albedo calculation was used for this calculation.

## Model results

The optimal hyperparameters for the exponential weighting function were a length scale of 16 pixels (160 m) for NDVI, and a length scale of 7 pixels (70 m) for albedo, for a window size of 51 $\times$ 51 pixels (250 m away from each center point). Using these hyperparameters, we apply a bootstrapping approach to estimate coefficients and confidence intervals (further details provided in the “Methods” section). Bootstrapped results are shown in Table 1. When interpreting these results, it is important to remember that the data is normalized (see “Methods” section). Thus, a coefficient value of zero means that the model just predicts the mean temperature.

**Table 1:** Bootstrapped model coefficients.

Dataset	#	Term	Coefficient mean	95% confidence interval
NLCD, as percentage of 51 $\times$ 51 pixel window	1	11—open water	−0.07	(−0.98, 1.08)
	2	21—developed, open space	**2.60**	(1.70, 3.60)
	3	22—developed, low intensity	**2.08**	(1.45, 2.76)
	4	23—developed, medium intensity	**2.01**	(1.61, 2.41)
	5	24—developed, high intensity	0.39	(−0.47, 1.05)
	6	31—barren land	0.02	(−0.56, 0.63)
	7	41—deciduous forest	**1.00**	(0.46, 1.56)
	8	42—evergreen forest	**1.18**	(0.53, 1.87)
	9	43—mixed forest	0.67	(−0.05, 1.48)
	10	52—shrub	−0.25	(−0.97, 0.57)
	11	71—grassland, herbaceous	−0.04	(−0.60, 0.58)
	12	81—pasture	**0.78**	(0.02, 1.42)
	13	82—cultivated crops	0.00	(0.00, 0.00)
	14	90—woody wetlands	**1.33**	(0.53, 2.15)
	15	95—emergent herbaceous wetlands	0.63	(−1.11, 1.97)
NDVI	16	Point	− **0.37**	(−0.55, −0.12)
	17	Spatial	−**3.25**	(−4.50, −2.31)
Albedo	18	Point	−0.12	(−0.51, 0.40)
	19	Spatial	−**1.06**	(−1.61, −0.32)

Coefficients that do not include zero in their confidence intervals are bolded.

**Figure 4:**
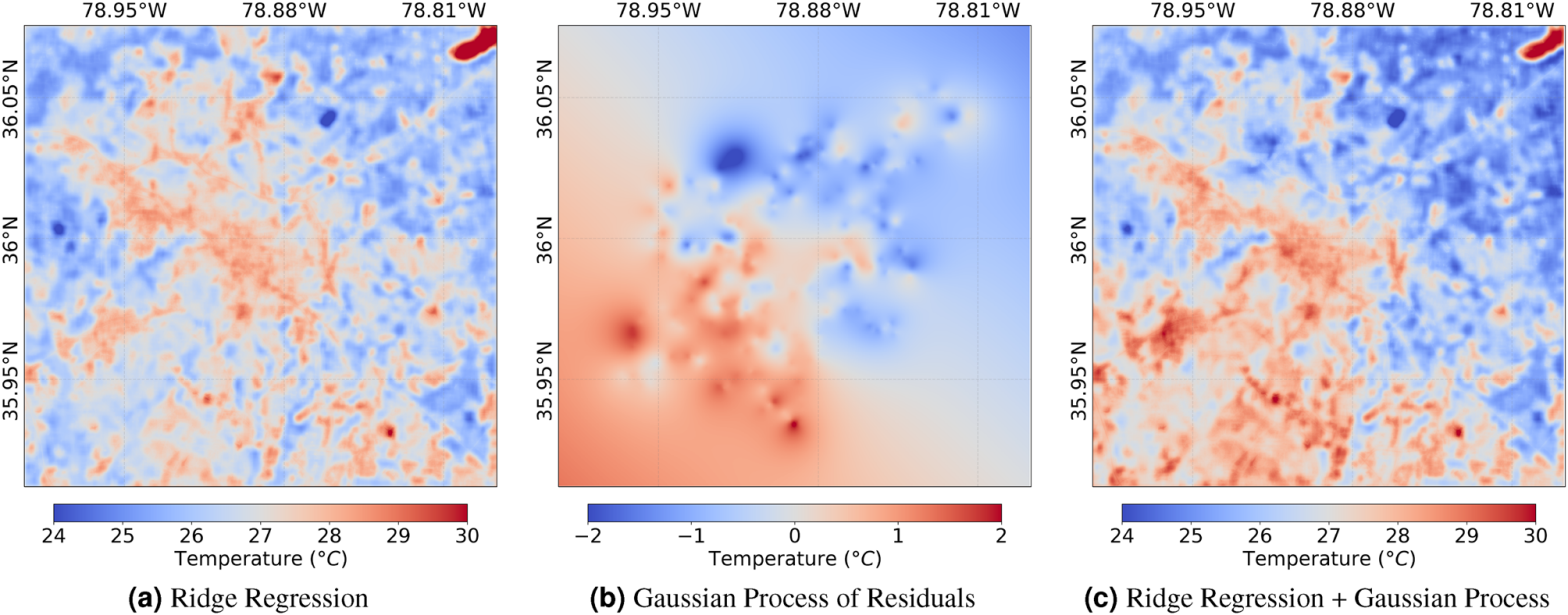
Temperature map before and after adding the unobserved spatial process.

To create the visuals in Fig. 4, we iterated between fitting the ridge regression and Gaussian process until the model converged on all traversal points, which yielded a final $R^2$ of 0.44 in the Ridge model. After fitting a Gaussian process to a sample of the residuals of this model, the $R^2$ of the combined Ridge-Gaussian process model on all of the data was 0.94. We use a sample of the residuals (1000 data points) to reduce the computational burden of the Gaussian process.

### Intervention analysis

This model can now provide us with insight into how expected interventions may reduce temperatures in urban heat islands. In this section, we propose three common interventions: lining streets with trees, adding in a park, and increasing the albedo of a building. We demonstrate how these interventions may be visualized and their corresponding direct, indirect, and overall effects on temperature calculated. These estimands are further defined in the “Methods” section.

We emphasize that these example interventions consider local effects on temperature when going from one extreme to another. That is, when going from no vegetation to vegetation, or from low albedo to high albedo. However, with the model trained, the interventions could easily be considered at specific locations. This is done by taking the treatment data source (i.e., NDVI), and altering pixel values at the location of interest, then comparing model outputs before and after the simulated intervention. Interventions may still be considered at the city level by comparing model outputs before and after implementing hypothetical policies. For example, one may consider the impact of increasing NDVI in all locations with an NDVI value below 0.2 to be 0.3. This would correspond to a policy in which places with little vegetation are targeted for greening initiatives.

#### Lining a street with trees

**Figure 5:**
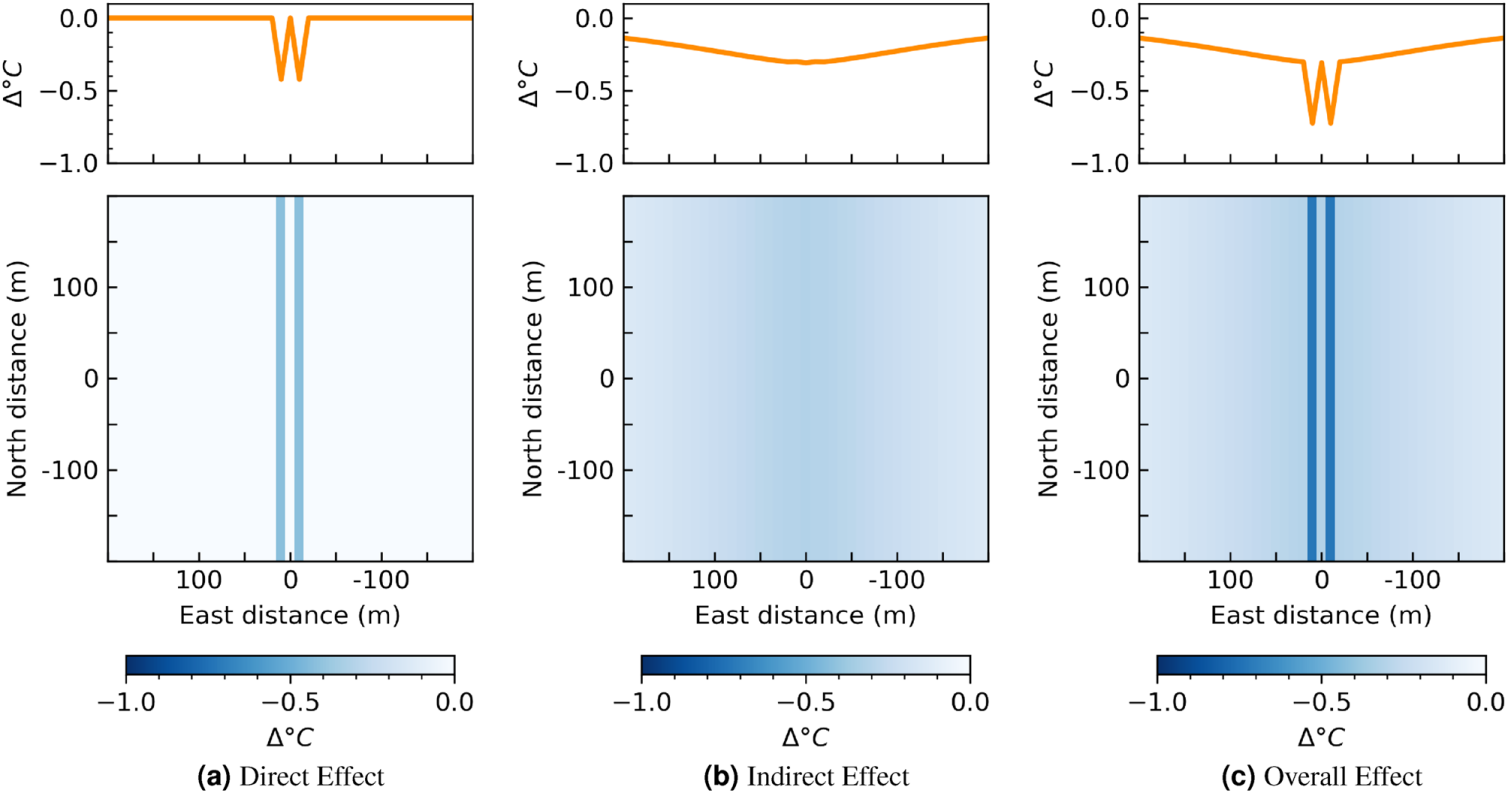
Temperature change for a street lined with trees modeled by changing NDVI from 0 to 1 along two parallel lines. Plots on top show effect on temperature as a function of distance from the intervention. Plots on bottom visualize the effect over space. The effect is quantified using $\Delta ^\circ C$, to suggest that this would be the change to temperature on average on any street where this intervention was implemented.

A common intervention is to line streets with trees. We may consider this effect through a pixel-wise change in NDVI from 0 to 1 along two parallel lines. The physical interpretation of this intervention is that there is a 30 m wide street canyon with 10 meter wide trees lining a 10 m wide street. We are restricted to considering 10-m by 10-m sized interventions due to the resolution of the data. We justify this size intervention because 10 m is approximately the canopy diameter of several common urban tree species, measured at maturity^35^. We visualize this effect in Fig. 5.

#### Replacing an urban block with a park

**Figure 6:**
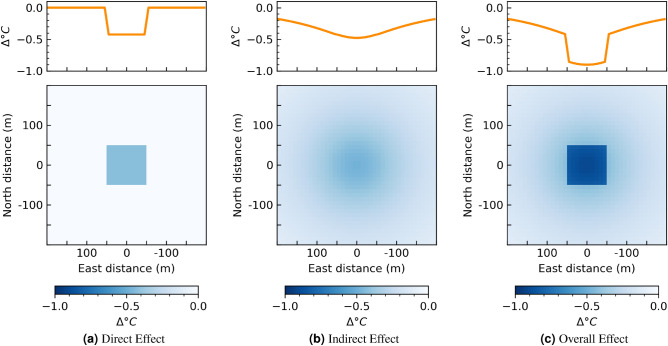
Temperature change estimated for a 100 m by 100 m park, with an NDVI of 1 within the park, compared to an NDVI of 0. Plots on top represent change in temperature as a function of the distance from the center of the intervention. Plots on bottom show the effect over space. This intervention represents an average effect for a park of this size, if it were to be implemented anywhere within the study area.

Again, we can consider the impact of greenspace on urban temperatures by looking at the impact of a park on temperatures, an intervention that has been richly studied in the past^36^. In this example, we can visualize the impact of a 100-m by 100-m park on temperature in Fig. 6. Compared with the previous example, the indirect effect is much larger, which suggests that larger patches of greenspace will have a greater indirect effect, and that we may expect larger green spaces to affect temperatures at further temperatures away.

#### A cool roof implementation

**Figure 7:**
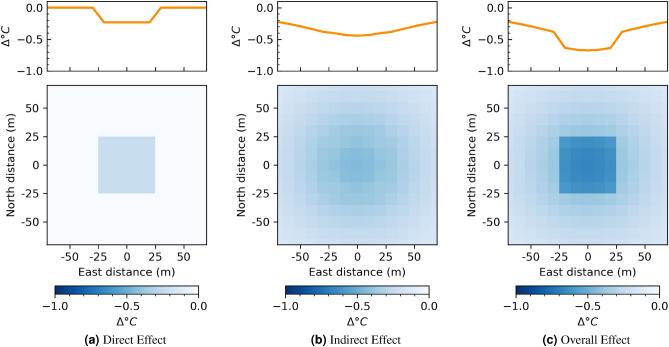
Temperature change estimated for a 50 by 50 m building, from an albedo of 0 to an albedo of 1. Plots on top are change in temperature along North distance 0, to show change in temperature as a function of distance from center of intervention. Plots on bottom show the areal effect. We note that this represents the average spatial effect of changing albedo in the study area.

Lastly, we may consider the effect of cool roofs with a similar visual to the example above—a simple increase in albedo from a low value (0) to a high value (1). This intervention is shown in Fig. 7.

## Discussion

The results above demonstrate that a causal inference based model can help decision makers understand both the magnitude and spatial extent of interventions on temperature.

The methodology presented in this work demonstrates how causal inference techniques can provide estimates of local treatment effects. This contribution is in contrast with previous works that calculate interventions at the city-wide scale. A local intervention approach should be preferred over city-wide analyses because interventions intrinsically take place at local scales, and are neither reasonable nor cost-effective when considered over the scale of an entire city.

This work is limited to analyzing temperature on a single day, during a single time period for that day. We admitted this limitation due to the desire to use higher spatial resolution air temperature data than previous studies. To avoid this limitation in the future, meteorological networks should expand. Indeed, tools such as Weather Underground are enabling this growth in data collection, so that future work can capture spatiotemporal variations in treatment effects. Furthermore, we restrict our analysis of interventions to that of vegetation and increasing albedo. This is because these interventions are the most often cited interventions in the literature. However, more complex interventions exist. For example, at the building scale, energy efficiency measures such as improving the building envelope reduce anthropogenic heating by decreasing the amount of heat expelled by air conditioning. To analyze the effect of such interventions on urban heat islands, the contribution of anthropogenic heating to the surface energy balance must be adequately characterized. Recent research has attempted to capture this relationship, and future work should analyze the ability of interventions to reduce the contribution of anthropogenic heating to urban heat island development^37^.

It is important to consider the limitations of this approach in contrast to alternatives (e.g., CFD, UCM, and black-box approaches). While our approach is less computationally intensive than these alternatives, it is restricted to learning spatially-averaged causal effects over the time period of the data. In contrast, alternative approaches can simulate the effects of interventions under diverse meteorological conditions. Furthermore, there is an inherent trade-off in expertise required to implement our approach over alternatives. While CFD and UCM require subject matter expertise in physical modeling, this approach requires greater expertise in the fields of spatial statistics and causal inference. This is often the case when considering data-driven methods for modeling the physical world. Despite these trade-offs, our approach provides a first-order approximation of intervention effects at the time of data collection. Since our data was collected on a prototypical warm summer day, we believe this estimate allows for a cheaply acquired understanding of the effects of interventions when they would be most important (i.e., during periods of extreme heat). Furthermore, our approach is advantageous in the absence of high resolution canopy data needed for accurate CFD or UCM approaches, since we rely instead on widely available satellite data. Future iterations of our approach may be more easily adopted by stakeholders with greater data collection and model refinement, as estimates may then be acquired for locations of interest with minimal or no further model refinement.

There are multiple future methodological improvements possible for this line of research. First, the model parameterized in this work assumes constant, isotropic interference within the location of interest, but it is reasonable to assume that the level of interference will vary across space and time, as a function of urban morphology and meteorological conditions. Intuitively, we would expect that interference will be lower over rougher surfaces (under weak synoptic conditions), as there will be greater vertical mixing. So, the highly urbanized regions may experience less interference. Indeed, several studies describe how mitigation effects vary over space and time^36^. Furthermore, this is suggested in our model, as the indirect effect of changing albedo is best modeled using a smaller length scale than the optimal length scale found for modeling NDVI. We believe this is because albedo is highest in urbanized settings that already use cool roofs (see Fig. 3d), so the length scale learned for albedo is biased to better represent urbanized effects, whereas NDVI is more variable over the whole area. Because of this, the model learns that changing NDVI has a larger indirect effect on average, when in fact that could be an artifact of the training data. Thus, future modeling approaches should consider spatiotemporally heterogeneous interference. Second, we assume a linear relationship between vegetation, albedo, and land use with temperature, when a non-linear relationship may be more appropriate. Certain interventions may lead to non-linear decreases in temperature through multiple physical changes to the environment. For example, replacing a parking lot with trees provides shade, alters the net radiation flux, and increases the amount of heat released as latent rather than sensible heat (i.e., more heat is used to evaporate water rather than heat air). Because there are multiple pathways of temperature reduction, we might expect the size of the treatment effect to vary non-linearly as a function of the intervention extent and existing local conditions. Lastly, this methodology has been developed to be easily applied to new locations. The data sources are all readily retrieved using Google Earth Engine, the National Land Cover Database, and NOAA’s urban heat island campaign. We expect that incorporating data from more cities will enable a model to learn heterogeneous interference levels and non-linear treatment effects.

The goal of this research is to demonstrate how spatially aware causal inference can empower precision climate management by informing decision makers of the ability of local interventions to reduce ambient temperature. With continued research into precision climate management, we can build more resilient cities to reduce heat risk, improve human comfort, lower energy demand, and decrease greenhouse gas emissions.

## Methods

### Data regularization

Prior to model fitting, all covariates were normalized to have a minimum of 0 and maximum of 1, so that we could apply L2 regularization during model fitting. Because of multicollinearity within these data sources (e.g., highly urbanized land cover classes tend to have a lower NDVI value), L2 regularization ensures that the model places greater weight on stronger relationships. Since the covariates had non-normal distributions that are heavily skewed in some cases, minimum/maximum standardization is preferred. Conversely, the outcome variable, temperature, followed an approximately Gaussian distribution, so we normalized this variable to have a mean 0 and standard deviation 1.

### Model training and hyperparameter tuning

To fit this model, we need to jointly train both the coefficients corresponding to the covariates, as well as the unobserved confounding variable. To do so, we iterated between training the model on the covariates using ridge regression and fitting the residuals using a Gaussian process. The initial ridge fit trains on the outcome variable, and in subsequent iterations the fit residuals from the Gaussian process are subtracted from the outcome variable, so that the regression learns model coefficients that have been deconfounded by the Gaussian process. This algorithm is summarized formally in Algorithm 1, in which we denote the log-likelihood of ridge regression as $\mathscr{L}$. Recall from earlier that we defined the covariance function for the Gaussian process as *K*, and that we treat the residuals as noisy. In the algorithm, the covariance function corresponds to a matrix of covariance values between observed data points. To incorporate noise into model fitting, a noise term $\sigma _n$ is added to the diagonals of this covariance matrix. This noise term is an additional hyperparameter to be learned. We found that model coefficients and performance tended to stabilize after 3–5 iterations, yet we iterate ten times so that we can ensure model convergence (in the algorithm, the iteration is denoted by *m*).

**Algorithm 1:**
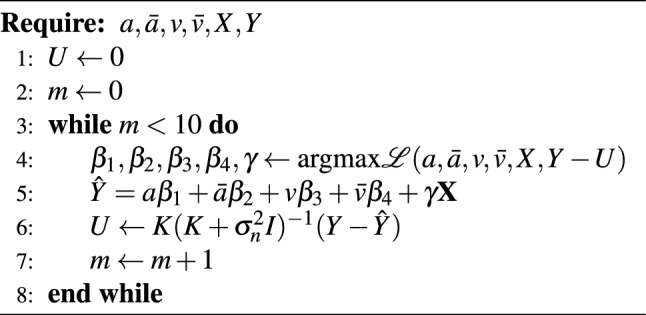
Training algorithm for ridge regression and Gaussian process.

**Figure 8:**
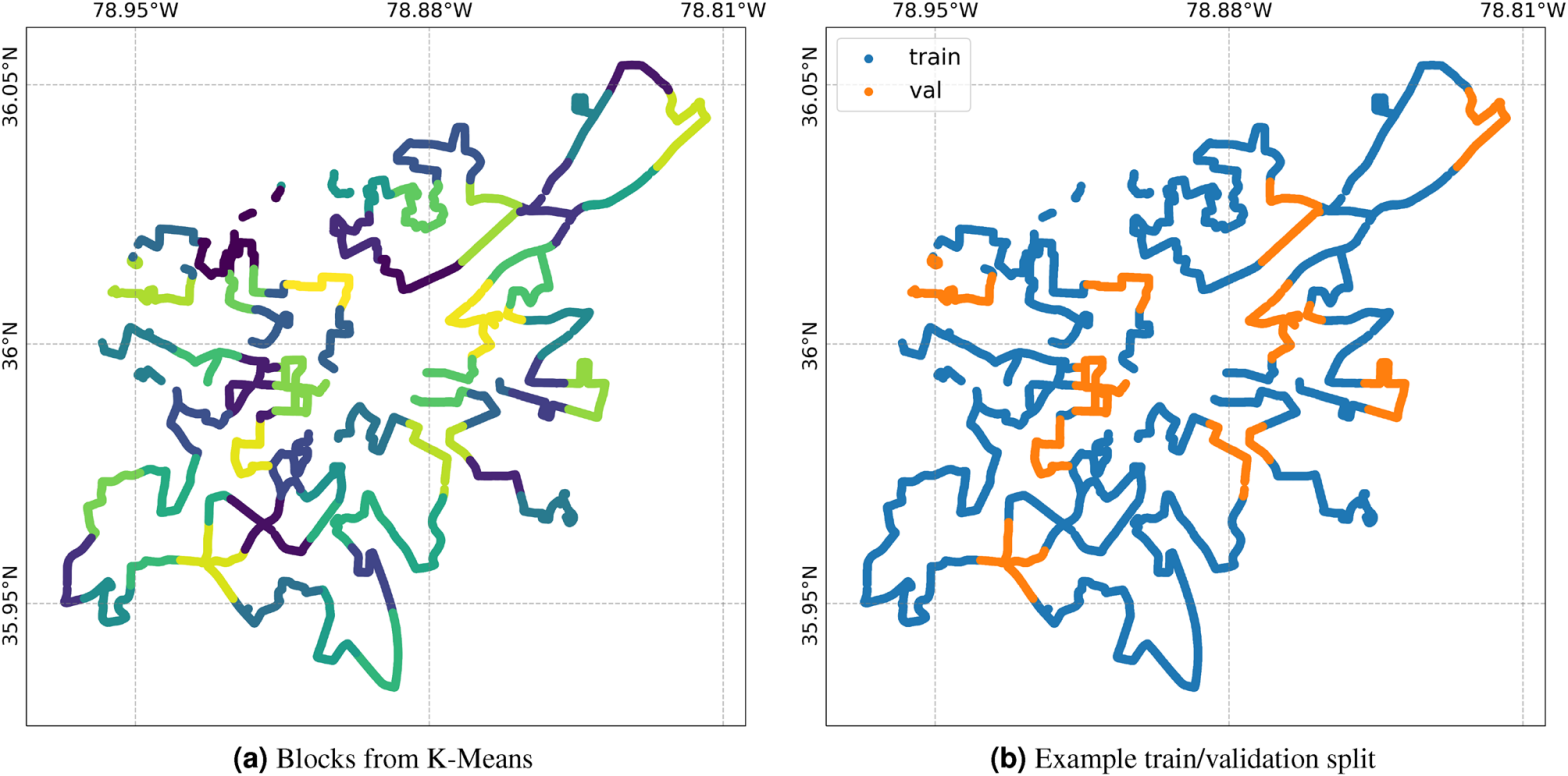
How K-means is applied to generate training and validation splits, and to apply block bootstrapping. The left map shows 50 splits of the data. The right map shows an example split, in which 40 blocks are used for training, and 10 blocks are used for validation.

To fine-tune the hyperparameters for ridge regression, the Gaussian process, and the length scale parameters for weighting functions $w_v$ and $w_a$, we perform a grid search employing a *k*-fold block validation strategy. The data points are first split into *k*-folds using K-means on the coordinates, so that the data is split into approximately *b* equal area blocks, according to the method outlined in^38^. We then get *k* folds from the blocks by selecting *b*/*k* blocks to include in the validation set, with the remaining blocks comprising the training set, as visualized in Fig. 8. This provides us with *k* splits of the data from which we can select the optimal hyperparameters based on the validation $R^2$ value of the ridge regression model, which we obtain by averaging the ridge regression validation performance over iterations 5–10. This block cross-validation strategy is important so that the model generalizes beyond learning the optimal weighting function that best captures autocorrelation, to learn causal relationships instead that capture the level of interference^39^. It is worth noting that using a grid search for Gaussian process hyperparameters is computationally expensive. In future iterations of this approach, we suggest applying gradient descent to these hyperparameters, as described in^40^.

To develop the algorithm above, we used the scikit-learn implementations of ridge regression, Gaussian processes, and K-means^41^.

### Uncertainty quantification

To calculate uncertainty of model parameters, we employ a block bootstrap approach. Similar to the k-fold validation strategy employed above, we again get *b* equal area blocks using K-means, then we sample *b* blocks, with replacement, to get a bootstrapped sample of the data (see Fig. 8b). We then perform the iterative procedure to fit the model, then save the model coefficients at iteration 5. This is performed over 100 different bootstrapped samples, so that we may obtain an empirical distribution function for each of the model coefficients. Given this cheap posterior estimate of model coefficients, we may then calculate a distribution for the causal estimands of interest^42^.

### Estimating the effects of interventions

In traditional causal inference, effects are often given as average treatment effects, wherein the effect of a treatment is estimated as the averaged difference between treated units and control units. In the presence of interference, it is necessary to characterize intervention effects using alternative causal estimands. While we refer the reader to^16^ for a more complete explanation of these estimands, we summarize these estimands in Eqs. (15)–(18). The direct effect, given in Eq. (15), allows us to estimate the effect of implementing an intervention at a specific point of interest, while keeping the neighboring effects constant.

15 $$\begin{aligned} DE_{ij}(\Delta a_{ij}, \bar{a}_{ij}) = \mathbb {E}[Y_{ij}(a_{ij} + \Delta a_{ij}, \bar{a}_{ij}) - Y_{ij}(a_{ij}, \bar{a}_{ij})] \end{aligned}$$

The indirect effect (Eq. (16)), calculates the effect of implementing an intervention in a local area, in which the point of interest is unchanged. In other words, this estimand calculates the effect of neighboring interventions when there is no change at point *ij*.

16 $$\begin{aligned} IE_{ij}(a_{ij}, \Delta \bar{a}_{ij}) = \mathbb {E}[Y_{ij}(a_{ij}, \bar{a}_{ij} +\Delta \bar{a}_{ij}) - Y_{ij}(a_{ij}, \bar{a}_{ij})] \end{aligned}$$

The total effect and overall effect (Eqs. 17 and 18, respectively), calculate the change at a point of interest when an intervention is implemented at both that point and in the surrounding area. The distinction between the two is that the total effect is used when there is an intervention at a point of interest, and this need not be the case for the overall effect. For our purposes, we are interested in understanding this overall effect, as we would like to understand the effect of interventions at both the points of intervention, and in the surrounding area.

17 $$\begin{aligned} TE_{ij}(\Delta a_{ij}, \Delta \bar{a}_{ij}) = DE(\Delta a_{ij}, \bar{a}_{ij}) + IE(a_{ij}, \Delta \bar{a}_{ij}) = \mathbb {E}[Y_{ij}(a_{ij} +\Delta a_{ij}, \bar{a}_{ij} +\Delta \bar{a}_{ij}) - Y_{ij}(a_{ij}, \bar{a}_{ij})] \end{aligned}$$

To make the distinction between the Total Effect and Overall Effect more clear, we denote that we are comparing an area after an intervention *a* with the pre-intervention area $a'$. Because we are looking at an area, and not just a specific pixel where an intervention took place, there might be some pixels in which no intervention took place.

18 $$\begin{aligned} OE_{ij}(a, a') = \mathbb {E}[Y_{ij}(a_{ij}, \bar{a}_{ij}) - Y_{ij}(a_{ij}', \bar{a}_{ij}')] \end{aligned}$$

## Data Availability

The datasets used are all publicly available, and code used for processing the data is provided in the code repository. Additionally, the post-processed datasets may be found in the code repository. Temperature data can be found at https://www.heat.gov/pages/nihhis-urban-heat-island-mapping-campaign-cities. Land cover data for 2021 can be accessed at https://www.mrlc.gov/data/nlcd-2021-land-cover-conus. Sentinel-2 satellite data for NDVI and albedo can be accessed using Google Earth Engine^34^.

## Code availability

The code used for this analysis may be found at https://github.com/zcalhoun/causal-uhi. The code is additionally being uploaded to the Duke University Digital Research Data Repository to guarantee long-term access and provenance with a permanent DOI.

## References

[1] Oke TR, Mills G, Christen A, Voogt JA (2017). Urban Climates.

[2] Imhoff ML, Zhang P, Wolfe RE, Bounoua L (2010). Remote sensing of the urban heat island effect across biomes in the continental USA. Remote Sens. Environ..

[3] Khatana SAM, Werner RM, Groeneveld PW (2022). Association of extreme heat with all-cause mortality in the contiguous US, 2008–2017. JAMA Netw. Open.

[4] Huang K (2021). Persistent increases in nighttime heat stress from urban expansion despite heat island mitigation. J. Geophys. Res. Atmos..

[5] Mora C (2017). Global risk of deadly heat. Nat. Clim. Change.

[6] Zhang B, di Xie G, xi Gao J, Yang Y (2014). The cooling effect of urban green spaces as a contribution to energy-saving and emission-reduction: A case study in Beijing, China. Build. Environ..

[7] Stone B (2014). Avoided heat-related mortality through climate adaptation strategies in three US cities. PloS ONE.

[8] Akbari H (2016). Local climate change and urban heat island mitigation techniques—The state of the art. J. Civ. Eng. Manag..

[9] Li D, Bou-Zeid E, Oppenheimer M (2014). The effectiveness of cool and green roofs as urban heat island mitigation strategies. Environ. Res. Lett..

[10] Rawat M, Singh R (2022). A study on the comparative review of cool roof thermal performance in various regions. Energy Built Environ..

[11] Bowler DE, Buyung-Ali L, Knight TM, Pullin AS (2010). Urban greening to cool towns and cities: A systematic review of the empirical evidence. Landsc. Urban Plan..

[12] Mirzaei PA (2015). Recent challenges in modeling of urban heat island. Sustain. Cities Soc..

[13] Adilkhanova I, Ngarambe J, Yun GY (2022). Recent advances in black box and white-box models for urban heat island prediction: Implications of fusing the two methods. Renew. Sustain. Energy Rev..

[14] Goldblatt R (2021). Remotely sensed derived land surface temperature (LST) as a proxy for air temperature and thermal comfort at a small geographical scale. Land.

[15] Song Y, Wu C (2018). Examining human heat stress with remote sensing technology. GIScience & Remote Sens..

[16] Reich BJ (2021). A review of spatial causal inference methods for environmental and epidemiological applications. Int. Stat. Rev..

[17] Holland PW (1986). Statistics and causal inference. J. Am. Stat. Assoc..

[18] Frangakis CE, Rubin DB (1999). Addressing complications of intention-to-treat analysis in the combined presence of all-or-none treatment-noncompliance and subsequent missing outcomes. Biometrika.

[19] Imbens GW, Rubin DB (2015). Causal Inference for Statistics, Social, and Biomedical Sciences.

[20] Wilf HS (2005). Generating Functionology.

[21] Papadogeorgou, G. & Samanta, S. Spatial causal inference in the presence of unmeasured confounding and interference. Preprint arXiv:2303.08218 (2023).

[22] Williams CK, Rasmussen CE (2006). Gaussian Processes for Machine Learning.

[23] Paciorek CJ (2010). The importance of scale for spatial-confounding bias and precision of spatial regression estimators. Stat. Sci. Rev. J. Inst. Math. Stat..

[24] Shandas V, Voelkel J, Williams J, Hoffman J (2019). Integrating satellite and ground measurements for predicting locations of extreme urban heat. Climate.

[25] North Carolina State Climate Office. *Urban Heat Island Temperature Mapping Campaign*. https://climate.ncsu.edu/research/uhi/ (2021).

[26] Chandler TJ (1962). Temperature and humidity traverses across London. Weather.

[27] Chun B, Guldmann JM (2014). Spatial statistical analysis and simulation of the urban heat island in high-density central cities. Landsc. Urban Plan..

[28] Almeida CR, Teodoro AC, Gonçalves A (2021). Study of the Urban Heat Island (UHI) using remote sensing data/techniques: A systematic review. Environments.

[29] Stewart ID (2011). A systematic review and scientific critique of methodology in modern urban heat island literature. Int. J. Climatol..

[30] GDAL/OGR contributors. *GDAL/OGR Geospatial Data Abstraction software Library*. 10.5281/zenodo.5884351 (Open Source Geospatial Foundation, 2023).

[31] Dewitz, J. *National Land Cover Database (NLCD) 2021 Products*. 10.5066/P9JZ7AO3 (2023).

[32] Stewart ID, Oke TR (2012). Local climate zones for urban temperature studies. Bull. Am. Meteorol. Soc..

[33] Bonafoni S, Sekertekin A (2020). Albedo retrieval from Sentinel-2 by new narrow-to-broadband conversion coefficients. IEEE Geosci. Remote Sens. Lett..

[34] Gorelick, N. *et al.* Google Earth Engine: Planetary-scale geospatial analysis for everyone. *Remote Sens. Environ.* (2017).

[35] Pretzsch H (2015). Crown size and growing space requirement of common tree species in urban centres, parks, and forests. Urban For. Urban Green..

[36] Doick KJ, Peace A, Hutchings TR (2014). The role of one large greenspace in mitigating London’s nocturnal urban heat island. Sci. Total Environ..

[37] Wang Y, Li Y, Sabatino SD, Martilli A, Chan PW (2018). Effects of anthropogenic heat due to air-conditioning systems on an extreme high temperature event in Hong Kong. Environ. Res. Lett..

[38] Ruß, G. & Brenning, A. Data mining in precision agriculture: Management of spatial information. In *Computational Intelligence for Knowledge-Based Systems Design: 13th International Conference on Information Processing and Management of Uncertainty, IPMU 2010, Dortmund, Germany, June 28–July 2, 2010. Proceedings 13*. 350–359 (Springer, 2010).

[39] Roberts DR (2017). Cross-validation strategies for data with temporal, spatial, hierarchical, or phylogenetic structure. Ecography.

[40] Jiang, Z., Zheng, T., Liu, Y. & Carlson, D. Incorporating prior knowledge into neural networks through an implicit composite kernel. Preprint arXiv:2205.07384 (2023).

[41] Pedregosa F (2011). Scikit-learn: Machine learning in Python. J. Mach. Learn. Res..

[42] Hastie, T., Tibshirani, R. & Friedman, J. *The Elements of Statistical Learning: Data Mining, Inference, and Prediction. Springer Series in Statistics* (Springer, 2009).

